# The Therapeutic Application of *Tamarix aphylla* Extract Loaded Nanoemulsion Cream for Acid-Burn Wound Healing and Skin Regeneration

**DOI:** 10.3390/medicina59010034

**Published:** 2022-12-23

**Authors:** Haiwad Gul, Rana Dawood Naseer, Ifraha Abbas, Ejaz Ali Khan, Habib Ur Rehman, Asif Nawaz, Abul Kalam Azad, Ghadeer M. Albadrani, Ahmed E. Altyar, Ashraf Albrakati, Mohamed M. Abdel-Daim

**Affiliations:** 1 Institute of Physiology and Pharmacology, University of Agriculture Faisalabad, Faisalabad 38000, Pakistan; 2 Department of Pharmacy, University of Agriculture Faisalabad, Faisalabad 38000, Pakistan; 3 College of Animal Science and Animal Medicine, Tianjin Agricultural University, Tianjin 300384, China; 4 Advanced Drug Delivery Lab, GCPS, Faculty of Pharmacy, Gomal University, D. I. Khan 29111, Pakistan; 5 Department of Pharmaceutical Technology, Faculty of Pharmacy, MAHSA University, Jenjarom 42610, Malaysia; 6 Department of Biology, College of Science, Princess Nourah bint Abdulrahman University, Riyadh 11671, Saudi Arabia; 7 Department of Pharmacy Practice, Faculty of Pharmacy, King Abdulaziz University, P.O. Box 80260, Jeddah 21589, Saudi Arabia; 8 Pharmacy Program, Batterjee Medical College, P.O. Box 6231, Jeddah 21442, Saudi Arabia; 9 Department of Human Anatomy, College of Medicine, Taif University, Taif 21944, Saudi Arabia; 10 Department of Pharmaceutical Sciences, Pharmacy Program, Batterjee Medical College, P.O. Box 6231, Jeddah 21442, Saudi Arabia; 11 Pharmacology Department, Faculty of Veterinary Medicine, Suez Canal University, Ismailia 41522, Egypt

**Keywords:** nanoemulsion, cream, *Tamarix aphylla*, wound healing, skin regeneration

## Abstract

*Background and Objectives:* Nanomedicine is a constantly growing field for the diagnosis and treatment of various diseases as well as for regenerative therapy. Nanotechnology-based drug-delivery systems improve pharmacological and pharmacokinetic profiles of plants based biologically active molecules. Based on traditional claims, leaves of the *Tamarix aphylla* (TA) were investigated for their potential healing activity on burn wounds. *Materials and Methods:* In this study, TA-based nanoemulsion was prepared. The nanoemulsion was characterized for size, zeta potential, pH, viscosity, and stability. The nanoemulsion containing plant extract was converted into cream and evaluated for its efficacy against acid-burn wounds inflicted in the dorsum of rabbits. The animals were classified into four main groups: Group A as a normal control group, Group B as a positive control (treated with cream base + silver sulfadiazine), Group C as a standard drug (silver sulfadiazine), and Group D as a tested (treated with nanoemulsion cream containing TA extract). The prepared system could deliver TA to the target site and was able to produce pharmacological effects. On days 0, 7, 14, 21, 28, and 35, wound contraction rate was used to determine healing efficacy. The wound samples were collected from the skin for histological examination. *Results:* Based on statistical analysis using wound-healing time, Group D showed a shorter period (21.60 ± 0.5098) (*p* < 0.01) than the average healing time of Group C (27.40 ± 0.6002) (*p* < 0.05) and Group B (33.40 ± 0.8126) (*p* < 0.05). The histopathological assessment showed that burn healing was better in Group D compared with Group C and Group B. The nanoemulsion cream had a non-sticky texture, low viscosity, excellent skin sensations, and a porous structure. By forming a protective layer on the skin and improving moisture, it enhanced the condition of burnt skin. *Conclusions:* According to the findings of this study, nanoemulsion cream containing TA extract has great potential in healing acid-burn wounds

## 1. Introduction

Implementation of nanotechnologies in diseases screenings, diagnosis and treatment, collectively stated as Nanomedicine, is an emergent field that can change person and population-based health in the 21st century [1]. Nanomedicine has opened new modules for wound healing by applying nanomaterial to deliver solutions to accelerate wound cure and demonstrate distinctive characteristics as bactericidal agents [2,3,4].

The nanoemulsions are clear, thermodynamically stable, oil–water dispersions stabilized by the interfacial coating by surfactant molecules with droplets smaller than 100 nm. The nanoemulsions have very low interfacial tension and considerable oil in the interfacial water area. The nanoemulsion has greater aspects of sustainability than basic micellar solutions, and their thermodynamic resilience gives them an edge upon unstable dispersions such as suspensions and emulsions since they are made with much less energy (heat or mixing) and have a long life span. Transport characteristics of the drug are influenced by the nano-sized droplets, resulting in large interfacial areas associated with nanoemulsions, which is a key feature in prolonged and targeted drug delivery [5]. The dermis and epidermis form a shield barrier against the surrounding environment on the skin surface. Once a wound is inflicted, the barrier is bleached [6]. Wound healing is a sequence of conjugate processes during which newly formed tissues replace the damaged tissues at the injury site. Wound healing consists of a five-step process involving hemostasis, neovascularization and fibroplasia, tissue granulation development, re-epithelialization, and finally, an emergence of a tissue remodeling and extracellular matrix [7].

Burn-wound infection remains a serious concern in burn centers, despite recent breakthroughs in burn treatment and management incorporating early excision and grafting. Infection in burn wounds is the leading source of mortality and morbidity, especially in the low- and middle-income nations [8]. There are three types of burns: first-degree (superficial) burn damage, mainly the skin’s surface layer; a color change (blackening of skin in black people and redness in whites), edema, discomfort, and dryness. These burns usually recover in three to six days; second-degree (partial thickness) burns: both the outer and inner layers are damaged, resulting in burning, swelling, and discomfort; most of these burns heal in two to three weeks, although some may take longer; third-degree (full thickness) burns: the skin is damaged to its entire thickness. Tendons and bones, important organs, and blood vessels might all be severely damaged. Due to nerve loss, pain in third-degree burns may go unnoticed. Charred, dark brown, waxy, elevated, and leathery burned areas are possible [9].

The significance of plants has been described in many Surahs of the Holy Quran, such as Al-Bakra, Al-Rehman, Al-Momeenoon, Ar-Rad, Al- Ibrahim, Al-Anaam, Al-Araf, and An-Nahl. Our Holy Prophet Hazrat Muhammad (SAW) utilized and advised therapeutic plants [10]. Plant origin remedies are used in traditional health care, and the World Health Organization has recognized their value in indigenous cultures. Natural medicinal herbs are easy to obtain, and these items are said to have fewer adverse effects [11,12]. Plants are one of the most significant sources of medicines and drugs today, and many plant products provide various secondary metabolites and essential oils [13]. Plants have long been used to treat various medical conditions. Plants are a key source of therapeutic preparations, as well as many medications developed from them [14,15]. 

TA is commonly known in English as tamarisk or athel and in Arabic as Ghaz or Athel, Abal, and Tarfaa. This plant was identified by Gustav Karl Wilhelm Hermann Karsten, a German botanist and geologist (*Tamarix aphylla* TSN 22306). TA is usually found in the form of a tree or tall shrub that grows up to twelve meters in height on alpine or alluvial land, and it has a dark red or grey bark. Almost all plants in the same family are found to grown successfully in subtropical and temperate regions. The halophytes can effectively tolerate a range of abiotic stress, such as drainage, salt, and temperature effects [16].

TA is the most widely cultivated plant in Pakistan [17]. Leaves of TA contain alkaloids, flavonoids, steroids, cardiac glycosides, saponins, terpenoids, and tannins [18]. Anti-inflammatory and wound soothing activity of the TA is also mentioned in the Islamic literature and from various sources out of the village areas of Saudi Arabia. The dry powder of TA from all parts of the herb was used in Al-Qassim in Saudi Arabia for the treatment of skin disorders of camel (allergic or mycological) when applied to the skin for a minimum of one week. The leaves of TA have been said to have germicidal properties. It is also used to treat eye irritation, fever, toothache (dental discomfort), wound healing, inflammation, and the common cold and flu [19,20]. TA leaves have been used for various ethno botanical and pharmaceutical purposes, including antioxidant action, usage in jaundice, rheumatism, bad evils, wounds, and abscesses [21]. In Pakistan, TA leaves are being using traditionally for recovery from skin injury or damage in Lakki Marwat district, Khyber Pakhtunkhwa. This study motivated further research on TA leaves because of the significant outcome from traditional uses and support by the scientific data. TA leaves when heated in water and attached to affected skin work wonderfully for wound treatment, rheumatism, and abscesses [22]. The objective of this study was the preparation and characterization of nanoemulsion cream and to evaluate the therapeutic efficacy of TA containing nanoemulsion cream in the treatment of acid-burn wounds. 

## 2. Materials and Methods

### 2.1. Materials

Silver sulfadiazine (Quench Cream) was used as standard medicine. Sulfuric acid and methanol were purchased from Merck KGaA (Merck KGaA, Frankfurter Strasse 250 64293 Darmstadt, Germany). Chitosan, tween 80, span 60, soft white paraffin, and liquid paraffin were used in nanoemulsion formulation and obtained from Sigma Aldrich(Sigma Aldrich, 14508 Saint Louis, MO 63178 USA). All the chemicals were of analytical grade. 

### 2.2. Collection and Extraction of Plant 

Plant leaves were collected in the month of December during its fruit season at District Lakki Marwat, KP, and Pakistan and were identified by Prof. Dr. Mansoor Hameed, Chairman Department of Botany, University of Agriculture Faisalabad, and Punjab, Pakistan. The leaves of the plant were dried at ambient temperature for three weeks. An electric grinder was used to grind the plant’s dried leaves into powder. 300 g of plant leaves powder was steeped in conical flasks having 200 mL of 70% methanol and kept in a horizontal shaker for three days. After shaking, the extract was filtered with the help of Whatman No.1 filter paper to remove insoluble particles. After the filtration, the extract was stored at room temperature. The methanol soluble residue obtained was concentrated with a rotary evaporator at 40 °C [23].

### 2.3. Qualitative and Quantitative Analysis of Plant Leaves

The extract was subjected for the quantitative and qualitative analysis of the various phytoconstituents using high-performance liquid chromatography (HPLC), total flavonoids test, total phenolic contents, and the antioxidant activity [24].

#### 2.3.1. High Performance Liquid Chromatography

The parameters and specifications of high performance liquid chromatography are as follows:

ParametersSpecificationsColumnShim-pack CLC-ODS (C-18), 25 cm × 4.6 mm, 5 µmMobile phase: Gradient:A (H_2_O: Acetic acid-94:6, PH = 2.27), B (Acetonitrile 100%), 0–15 min = 15%B, 15–30 = 45%B, 30–45 = 100%BFlow Rate1 ml/minDetectionUV-Vis-280 nm and UV-Vis-248DetectorGradient HPLC and Isocratic HPLCModelSPD-10AVTemperatureRoom TemperaturePumpLC-10AT

#### 2.3.2. Determination of Total Phenolic Contents (TPC)

The Folin–Ciocalteu method evaluated the total phenolic content of TA leaf extract [25]. 1 mL of TA leaf extract were mixed with 5 mL Folin–Ciocalteu (10%) and four ml sodium carbonate (20%) then incubated for 1 h. The absorption of the resulting blue complex measured 765 nm. The solution was quantified following the standard gallic acid method. Different amounts of gallic acid were used to create the calibration curve. One ml aliquots of 0.01, 0.02, 0.03, 0.04, 0.05, 0.06, 0.07, 0.08, 0.09, and 0.10 mg/mL gallic acid solution in methanol were combined with 5 mL Folin–Ciocalteu reagent (diluted ten times) and 4 mL sodium carbonate (20%). After 1 h, the absorbance at 765 nm was measured, and the calibration curve was drawn using absorbance as a function of the concentration. The following method determined the total amount of phenolic compounds in plant extracts in gallic acid equivalents (GAE):

T = C × V/M

where,

T = total phenolic component concentration in mg GAE/g plant extract.

C = the gallic acid concentration in mg/mL derived from the calibration curve.

V = the volume of extract in ml.

M = the weight of plant leaf extracts in grams.

#### 2.3.3. Determination of Total Flavonoids Contents

Total flavonoid contents of plant extract were measured using the technique described by Chang [26]. In a 6-min incubation period, 0.5 mL of the extract was combined with 2 mL of distilled water and 0.15 mL of a 5% NaNO_2_ solution. After that, 0.15 mL of 10% AlCl_3_ solution was added to the mixture, and it was incubated for another 6 min before adding 4% of NaOH solution. The volume of the mixture was increased to 5ml by adding methanol and mixed well. After 15 min of incubation, the reaction mixture’s absorbance was measured at 510 nm. The extracts’ total flavonoid contents (TFC) were calculated using the catechin linear regression curve and represented as catechin equivalents.

#### 2.3.4. DPPH Scavenging Activity

The technique of Yen and Chen was used to measure DPPH radical-scavenging activity with minimal modification [27]. 3 mL of plant extracts at different concentrations were added to 1 mL of newly produced 0.004% DPPH in methanol solution, and the mixed solution was stored in the dark for thirty minutes. The absorbance was then measured at 517 nm. A reaction combination with low absorption shows significant radical-scavenging activity. The antioxidant activity of BHT and ascorbic acid, which served as a control, was also investigated. As a control, a solution without plant extract was utilized. All of the tests were performed three times. The following formula was used to compute the % inhibition of DPPH radical samples:

DPPH Inhibition (%) = Absorbance of Blank − Absorbance of Sample/Absorbance of Blank × 100.

### 2.4. Preparation of Nanoemulsion

Water in oil (W/O) emulsions were prepared by mixing different ratios of water, oil, and surfactants. The materials and quantity were presented at Table 1. Using a hot plate magnetic stirrer heated to 65 °C, an aqueous phase was produced by dissolving 3 g of leaf extract and 200 mg chitosan in a beaker holding distilled water. 7% *w*/*w* of Tween-80 was added when the extract and chitosan were completely dispersed in water.

For oil-phase preparation, 3.50 g of olive oil and 3% *w*/*w* of Span 60 were added to olive oil and heated to 65 °C using a hot plate magnetic stirrer. 

After preparing aqueous and oil phases, an aqueous phase was slowly added to the oil-phase beaker with continuously stirring over the hot plate magnetic stirrer. Finally, the nanoemulsion was formulated using a homogenizer (Diahan scientific Co., Ltd., GANGWON-DO Wonju-si 26358, Korea) one time at room temperature for 5 min at 10,000 rpm and 20 MPa pressure with 40% amplitude two times in each 2.5 min interval. The nanoemulsion produced was kept at 25 °C till further use.

### 2.5. Physiochemical Characterization of Nanoemulsion 

#### 2.5.1. Analysis of Size and Zeta Potential of Nanoemulsion

The size and surface charge of the nanoemulsion was determined by photon-correlated spectroscopy using a zeta sizer 1000 HS (Malvern Instruments Ltd. Enigma Business Park, Grovewood Road, Malvern, Worcestershire WR14 1XZ. UK). The nanoemulsion was taken in cell and then measured using a zeta sizer and the outcome was documented. 

#### 2.5.2. pH Determination 

The pH of formulated nanoemulsion was measured thrice at room temperature using a digital pH meter (lnolab pH 720). Before measuring the pH of nanoemulsion, the pH meter was calibrated with buffer solution. 

#### 2.5.3. Viscosity of Nanoemulsion 

The viscosity of the freshly prepared nanoemulsion was measured at room temperature using a Brookfield viscometer (BROOKFIELD ENGINEERING LABORATORIES, INC. 11 Commerce Boulevard, Middleboro, MA 02346 USA) fitted with a UL-adapter to assess its rheological characteristics.

#### 2.5.4. Skin Irritancy Test 

It was performed on the male albino rabbit weighing 1.5 kg having an age of 11 weeks. The animal was kept under standard laboratory conditions. A single dose of 10 mL of nanoemulsion cream was applied on the left ear, with the right ear as control.

#### 2.5.5. Stability of Nanoemulsion

The nanoemulsions should be considered a thermodynamically stable system produced by a combination of particular oil concentration, surfactants, and water. For one month, the stability of nanoemulsion was tested at four different temperatures: 5 °C, 15 °C, 25 °C, and 40 °C. Phase separation and cracking were both observed [28].

### 2.6. Preparation of Cream

The cream was prepared by mixing 18.50 g of soft white paraffin, 12 g of melted emulsifying wax, and 7 g of liquid paraffin. After this, 2 g of cetyl alcohol was dissolved in 10.5 g of water. Then both the solutions were added to a single beaker, stirred in a hot plate magnetic stirrer to 65 °C, and cooled to room temperature.

The stored nanoemulsion was mixed with the cream properly using a hot plate magnetic stirrer. Phase separation, cracking, and creaming were all observed for a month at 5 °C, 15 °C, 25 °C, and 40 °C. After the formation of nanoemulsion cream, it was stored in a well-closed container at room temperature. The concentration of extract in the final nanoemulsion cream was 30 mg/mL. 

### 2.7. Experimental Animals

The experimental trial was carried out with 20 albino rabbits having weights ranging from 1.4 to 1.8 kg and of age 10 to 12 weeks that were bought from the local market of Faisalabad. All of the rabbits were housed in the experimental animal house in an iron cage for about 14 days in the Institute of Microbiology, University of Agriculture Faisalabad before starting of the experimental trial to habituate to the environment. Each albino rabbit received two subcutaneous injections of ivermectin at the dose rate of 700 mcg/kg during the habitual period of after 1 week. Fresh and clean ad libitum water with the standard pellet was provided during the whole period of the trial. All the animals were used and cared according to rules defined by the Institutional Bioethics Committee, University of Agriculture Faisalabad.

#### 2.7.1. The Induction of Anesthesia and the Wound Infliction

The animals were shaved at the dorsum with the help of scissor and razor in antiseptic environment and were sanitized properly. After sanitization, the rabbits were anesthetized with the help of xylazine at the dose rate of 5 mg/kg of body weight and ketamine hydrochloride at the dose rate of 35 mg/kg of body weight injected intramuscularly in sternal recumbence. The 2 cm × 2 cm wound was created only on Group B, C, and D with the help of sulfuric acid by placing parchment paper and a prominent acid-burn wound of 2 cm × 2 cm was formed after 24 h. 

#### 2.7.2. Experimental Design and Treatment Protocol

The group was divided into four groups, each group contains 5 animals. The treatment interval was once a day.

Group A: Normal Control (Having no wound and normal food and drinking water)
Group B: Positive Control (Having acid-burn wound and treated with simple cream base and silver sulfadiazine + normal food and drinking water)
Group C: Drug control (Having acid-burn wound treated with standard drug (Silver sulfadiazine) + normal food and drinking water)
Group D: Treatment control (Having acid-burn wound treated with nanoemulsion cream containing TA extract + normal food and drinking water)

#### 2.7.3. Healing Time

Healing time was considered from the day of wound creation to re-epithelization. The healing time was estimated by the summation of everyday observation till the scars were off [29].

#### 2.7.4. The Wound Contraction Rate

It is the reduction in the original wound and is expressed as the wound contraction rate. The measurement of the wound was carried out on alternate days till the complete healing of the wound. The measurement was performed with the help of the Vernier caliper on alternate days until healing and was compared with the wound-creation day, that is, day 0.

The following formula was used to calculate the percentage of contraction [30]:

(1) $$\mathrm{Contraction}\;\%=\frac{\left[\mathrm{Area}\;\mathrm{on}\;\mathrm{day}\;\mathrm{zero}\;\left(\mathrm{mm}\right)-\mathrm{Area}\;\mathrm{on}\;\mathrm{day}\;\mathrm{measurement}\;\left(\mathrm{mm}\right)\right]}{\mathrm{Area}\;\mathrm{on}\;\mathrm{day}\;\mathrm{zero}\;\left(\mathrm{mm}\right)}\;\times 100$$

### 2.8. Histopathological Evaluation 

At the end of the trial, twenty male rabbits were anaesthetized to collect skin samples. After washing the samples with ice-cold normal saline (0.9 percent NaCl), the specimens were preserved in 10% formalin. The technique proposed by Bancroft and Gamble was used to conduct the histopathological examination of preserved tissue samples [31].

### 2.9. Statistical Analysis 

The data were then examined using the analysis of variance (ANOVA). Using the statistical computer software SPSS version 25.00, the means were compared using Tukey’s range test with a probability of 5%.

## 3. Results

The purpose of this study was to examine the therapeutic efficacy of nanoemulsion cream containing TA leaf extracts on acid-burn wounds. The research was carried out to determine the beneficial benefits of the plant TA on acid-burn wounds created on the dorsum of rabbits. Many plants and their products have previously been claimed to have considerable wound-healing properties. One of them is C. asiatica, a sweet, acrid, tropical weed/green useful in wound healing [32].

### 3.1. Qualitative and Quantitative Analysis of Plant Leaves

Leaf extracts of the TA were tested for total phenolic contents (TPC), total flavonoid contents (TFC), and 2,2-diphenyl-1-picrylhydrazyl (DPPH) to know about the antioxidant potential of the plant. Phenolic and flavonoid compounds are important constituents of the TA plant Table 2 and Table 3, and HPLC was performed for it in which quercetin was found as a flavonoid compound, while gallic acid, caffeic acid, vannilic acid, benzoic acid, syringic acid, p-coumaric acid, m-coumaric acid, and ferulic acid were found as phenolic compounds [33]. The total phenolic content of TA leaf extract was 220.6 mg/g gallic acid equivalents in mg GAE/100 g, while the TPC in TA leaves in literature is 262.26 mg/g [34]. The total flavonoid content in TA leaves was 69.60 mgQE/100 g, while the total flavonoid content in TA leaves in literature is 76.15 mgQE/100 g [35].

#### 3.1.1. High Performance Liquid Chromatography

**Figure 1:**
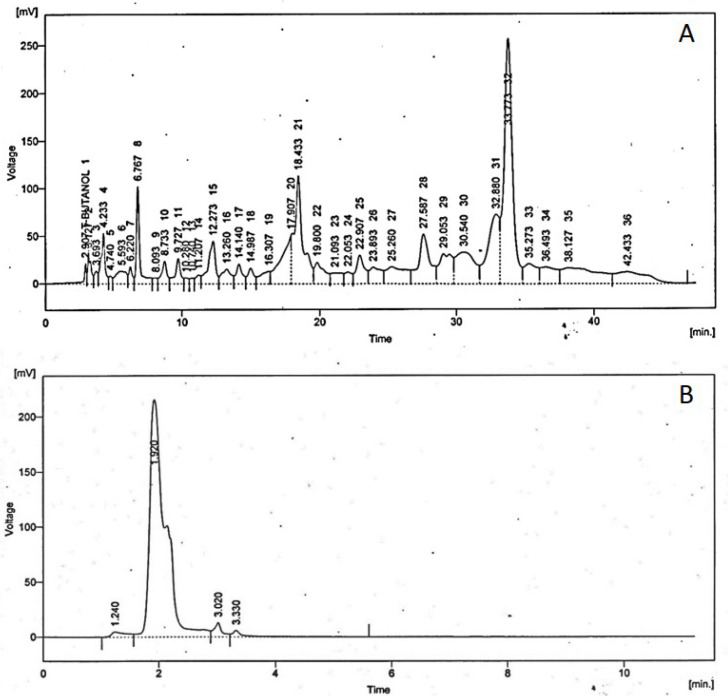
Chromatograph obtained by (**A**) gradient HPLC for analysis of flavonoids and phenolic acids and (**B**) isocratic HPLC for analysis of kaempferol in TA leaf extract.

High-performance liquid chromatography (HPLC) is the most frequently applied technique used for the analysis of phenolic acids and flavonoids in plant extracts. All the results are in ppm (parts per million) at Figure 1.

#### 3.1.2. Total Phenolic Contents (TPC)

Phenolic compounds are important plant constituents as they exhibit antioxidant activity by inactivating lipid free radicals or preventing the decomposition of hydroperoxides into free radicals. Plant phenolic extracts are always a combination of several phenol classes that are selectively soluble in various solvents. For the extraction, using an alcoholic solution yield shows good results; the optimum solvents for extracting phenolic components from TA leaves are aqueous alcohol solvents. The total phenolic content of TA leaf extract was 220.6 ± 2.8 mg/g gallic acid equivalents in mg GAE/100 g in Table 4. 

The use of a combination of alcohol and water has the benefit of modifying the polarity of alcohol solvents and the fact that polyphenol solubility is primarily determined by hydroxyl groups, molecular size, and hydrocarbon length.

#### 3.1.3. Total Flavonoid Contents (TFC) 

The total flavonoid contents (TFC) of the extracts were calculated using the catechin linear regression curve and represented as catechin equivalents. The total flavonoid content in TA leaves is 69.60 ± 1.5 mg QE/100 g in Table 5. 

#### 3.1.4. DPPH Scavenging Activity

The technique of Yen and Chen was used to measure DPPH radical-scavenging activity with minimal modification [36]. 3 mL of plant extracts at different concentrations were added to 1 mL of newly produced 0.004 percent DPPH in methanol solution, and the mixed solution was stored in the dark for 30 min. At 517 nm, absorbance was measured. The % inhibition of DPPH recorded is 39.014 ± 1.03% in Table 6. 

### 3.2. Hkh Characterization of Nanoemulsion

#### 3.2.1. Analysis of Size and Zeta Potential of Nanoemulsion 

**Figure 2:**
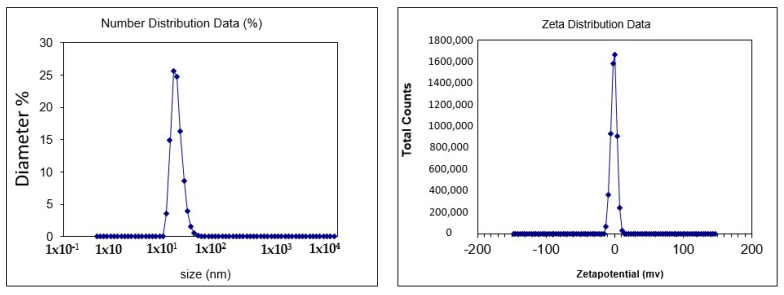
Shows the size and zeta potential of nanoemulsions.

The size of the nanoemulsion was analyzed with the help of a zeta sizer in Figure 2. Nanotechnology encourages the efficient use of chemicals that pose a threat due to low bio-absorption or high doses over short periods. The zeta size is a critical metric in nanoemulsion characterization. The zeta size of the produced nanoemulsion ranged from 1 to 275 nm in diameter. A zeta sizer is a type of equipment used to measure the size of nanoemulsions. Nano formulations having a size less than 200 nm are considered more efficient for the delivery of therapeutic agents. In recent years, research in the field of nanoscience has grown at an unparalleled rate. Nanotechnology used in medicine and surgery is expected to lead to major disease diagnosis, treatment, and prevention [37]. The zeta size of the prepared nanoemulsion was 13.3 nm. The zeta potential of the nanoemulsion was −1.09 mV, which compared with other nanoemulsion prepared for wound healing was smaller (29.10 ± 3.20 nm). Similarly, the zeta potential was also less than in the literature (−31.40) [38].

#### 3.2.2. pH of Nanoemulsion

The pH of formulated nanoemulsion of *Tamarix* aphyllla leaf extract was 5.7.

#### 3.2.3. Viscosity of Nanoemulsion 

The viscosity of the nanoemulsion recorded is 8.64 N.S/m².

#### 3.2.4. Skin Irritancy Test 

It was performed on the male albino rabbit weighing 1.5 Kg having an age of 11 weeks. The animal was kept under standard laboratory conditions. A single dose of 10 mL of nanoemulsion cream was applied on the left ear, with the right ear as control. There was no sign of edema, and Escher or erythema were observed on the ear of the rabbit. A skin irritation test was conducted for any hypersensitivity reaction before applying nanoemulsion cream containing TA leaf extract on wounds. A chemical is deemed an irritant when it produces reversible skin lesions such as inflammation that have healed partly or entirely until the observation period. Chemical can cause skin irritation, ulcers, bleeding, and body scabs in rabbits. This test showed the biocompatibility of nanoemulsion cream containing TA leaf extract with the skin, and it is non-immunogenic and safe.

#### 3.2.5. Stability of Nanoemulsion 

The nanoemulsions should be considered a thermodynamically stable system produced by a combination of particular oil concentration, surfactants and water. For one month, the stability of nanoemulsion was tested at four different temperatures: 5 °C, 15 °C, 25 °C, and 40 °C. During this phase, there was no separation, cracking, or creaming of nanoemulsion.

### 3.3. Healing Time

**Figure 3:**
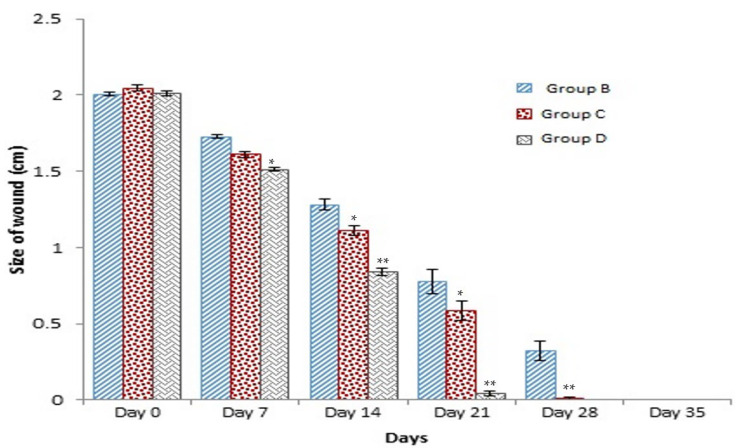
Shows time and wound healing progress in (cm), * indicate (*p* < 0.05) and ** indicate (*p* < 0.01) in statistical differences.

The healing time was calculated based on daily visual inspection and wound observation until the scars were off. The nanoemulsion cream containing TA leaf extract had a significantly different (*p* < 0.01) spectrum of healing of entire acid-burn wounds, and the frequency of therapy was significantly improved. Rabbits fared much better in the first two weeks, and healing improved in the TA group compared with the silver sulfadiazine group and control group (Figure 3, Table 7 and Table 8). The TA-treated group (Group D) started to improve the wounds from at day 7 onwards. It showed significant differences (*p* < 0.05) from group B but no statistical difference from group C. However, the data showed significant differences in recovering wounds between groups B and C (*p* < 0.05). The TA-treated group (Group D) had potential improvement at day 14, which created statistical differences of group D (*p* < 0.01) from both groups B and C. Figure 3 shows clear statistical differences (*p* < 0.01) among the groups based on wound improvement at days 21 and 28, but on day 35, all the groups were improved; the wounds in group B and C were treated with standard drug. 

### 3.4. Contraction Rate

**Figure 4:**
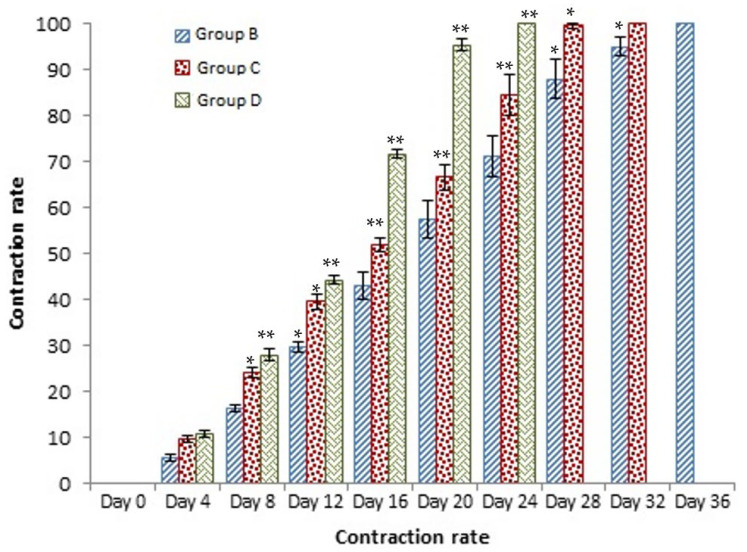
Shows the wound contraction rate against the time, * indicate (*p* < 0.05) and ** indicate (*p* < 0.01) in statistical differences.

It is the reduction in the original wound and is expressed as the wound contraction rate (Figure 4). The measurement of the wound was carried out on each day till the complete healing of the wound. Wounds were measured with the help of the Vernier calliper.

On day four, the wound contraction of the TA group is 10.75%, while that of silver sulfadiazine and the control group is 9.68% and 5.58%, respectively. On day 8, the wound contraction of the TA group is 27.96% (*p* < 0.01), while that of silver sulfadiazine and the control group is 24.14% (*p* < 0.05) and 16.25%, respectively. On day 12, the wound contraction of the TA group is 44.28% (*p* < 0.01), while that of silver sulfadiazine and the control group is 39.59% (*p* < 0.01) and 29.81% (*p* < 0.05), respectively. On day 16, the wound contraction of the TA group is 71.64% (*p* < 0.01), while that of silver sulfadiazine and the control group is 51.91% and 42.97%, respectively. On day 20, the wound contraction of the TA group is 95.32% (*p* < 0.01), while that of silver sulfadiazine and the control group is 66.57% and 57.43%, respectively. On day 24, the wound contraction of the TA group is 100%, while that of silver sulfadiazine and the control group is 84.46% and 71.29% (*p* < 0.01), respectively. On day 28, the wound contraction rate of silver sulfadiazine is 99.01% (*p* < 0.01) while that of control is 88.04% (*p* < 0.05). On day 32, the wound contraction rate of silver sulfadiazine is 100%, while that of control is 95.01%. The control wound contraction rate was 100% on day 36. The prepared nanoemulsion cream containing TA leaf extract was applied on the induced experimental animals compared with the commercial and untreated groups. Quench cream was used containing silver sulfadiazine as a commercial product. The size of the wounds was measured for 35 days, and wounds treated with nanoemulsion cream containing TA leaf extract showed remarkable healing activity. During the second week of the trial, the wound treated with nanoemulsion cream containing TA leaf extract was reduced up to 60%. This rapid activity of healing was due to nanoemulsion cream. TA leaves contain various phytochemicals that play a major role as an antioxidant that prevents oxidation, a chemical reaction that can lead to free radicals and chain reactions that can damage animals’ cells. After three weeks, that is 21 days, the acid-burn wound treated with nanoemulsion cream containing TA leaves showed complete healing, while the other acid-burn wounds treated with silver sulfadiazine showed complete healing after 28 days. Similarly, the wounds that were kept as control showed complete healing after 36 days. 

### 3.5. Histopathological Analysis

**Figure 5:**
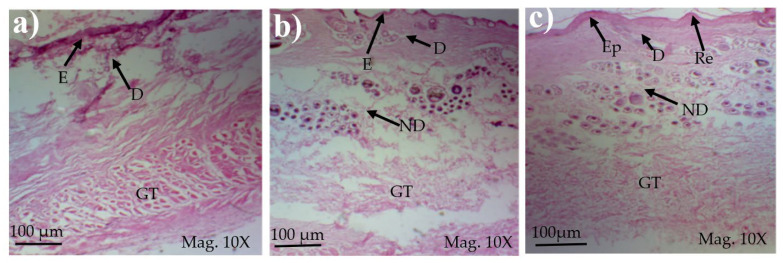
Healed Wound tissue treated with (**a**) Control; (**b**) Standard and (**c**) nanoemulsion, re-epithelialization (Ep) regeneration edge (Re), epidermis (E), dermis (D), normal dermis (ND) and granulation tissue (GT).

Parameters (epidermal thickness, collagen content, and thickness of dermis) were assessed during the histological examination using a tissue sample obtained at the wound site and analyzed in the histopathology laboratory after the study. The degree of compression and orientation of the collagen fibers were determined using percentages. For histopathological studies, the skin tissues of the affected rabbits were taken at day 35 and were dipped in formalin 10% solution for 72 h and later were processed for histopathology study. The histological studies (epidermal thickness, collagen content and thickness of dermis) showed that the nanoemulsion containing TA accelerated the wound-healing process better than the silver sulfadiazine. It was also revealed that nanoemulsion containing TA extract has significant cells attachment at the edge of the wound (Figure 5.)

#### 3.5.1. Thickness of Epidermis

When wounds treated with TA were compared with wounds treated with other treatments, it was discovered that wounds treated with TA had a thicker epidermis. Both treatment results were examined for epidermal thickness. According to the findings, the wounds treated with TA had a better epidermal thickness of 150.20 micron. In terms of epidermal thickness, the TA and silver sulfadiazine treatments outperformed the control. Mean table comparison showed that TA had the highest epidermal thickness in treatment of full thickness burn wounds.

#### 3.5.2. Collagen Content Percentage

Tissue samples were analyzed in the laboratory at 35th day. Compared with wounds treated with silver sulfadiazine and control, TA wounds had greater collagen content. In contrast to other treatments, wounds with no treatment featured loose collagen arrangements and minimal vascularization.

#### 3.5.3. Thickness of Dermis

Significant pain relief was achieved within 24 h, and overall healing period was achieved at twenty-one days compared with the positive control and standard groups Table 9.

After a detailed check, it was observed that TA had a thicker dermis than the other therapies employed in the study. Positive control wounds had less dermis thickness than other treatments. TA has been used for many testimonies since ancient times. Wound healing is one of the main indicated uses of TA in many countries [39,40]. The results of this study give a free hand to companies to start the formation of medicines in the form of ointments or cream. We hope a new burn ointment or cream can use herbal medicines with less unfavorable effects that will help in shortening the healing period, in turn decreasing the hypertrophic scar rate. Results showed that TA burn treatment is a cheap and useful herb. 

## 4. Discussion

The skin is the body’s largest organ, performing various vital activities such as homeostasis, immunity, nerve sensations, body temperature regulation, and metabolism. The skin also serves as a physical barrier, protecting the body from infection [41]. If this barrier is breached, microorganisms can enter the body and infect it directly. Inflammation, proliferation, and remodeling are the three phases in the injury healing process. Several components that might harm healing can disrupt the natural healing process at any stage. Pathological symptoms linked with diabetes, immune-related diseases, ischemia, venous stasis, frostbite, and general injury can all heal a damaged wound [42]. Epithelialization is the final step in the development phase. It includes migrating, proliferating, and differentiating epithelial cells in wound cells to repair abnormalities. To ensure epithelial cell migration with full-thickness open burns, epithelialization is postponed until a layer of granular tissue forms [43]. Burns are the leading cause of death in individuals with severe burns, according to several studies. As a result, numerous researchers have worked to find effective therapies to minimize the risk of wound infection and the length of time required to treat burn injuries. Topical antimicrobials, for example, are one of these therapies that successfully reduce burn mortality [44,45]. Nanotechnology encourages the efficient use of chemicals that pose a threat due to low bio-absorption or high doses over short periods. The zeta size is a critical metric in nanoemulsion characterization. The zeta size of the produced nanoemulsion ranged from 1 to 275 nm in diameter. A zeta sizer is a type of equipment used to measure the size of nanoemulsions. Nano formulations having a size less than 200 nm are considered more efficient for the delivery of therapeutic agents. In recent years, research in the field of nanoscience has grown at an unparalleled rate. Nanotechnology used in medicine and surgery is expected to lead to major disease diagnosis, treatment, and prevention [46]. The zeta size of the prepared nanoemulsion was 13.3 nm. The zeta potential of the nanoemulsion was −1.09 mV, which is smaller than other nanoemulsions prepared for wound healing (29.10 ± 3.20 nm). Similarly, the zeta potential was also less than literature (−31.40) [47]. The research was carried out to determine the beneficial benefits of the plant TA on acid-burn wounds created on the dorsum of rabbits. Many plants and their products have previously been claimed to have considerable wound-healing properties. One of them is C. asiatica, a sweet, acrid, tropical weed/green useful in wound healing [48].

Leaf extract of the TA were tested for TPC, TFC, and DPPH to know about the antioxidant potential of the plant. Phenolic and flavonoid compounds are important constituents of the TA plant. The total phenolic content of TA leaf extract was 220.6 mg/gm gallic acid equivalents in mg GAE/100 g, while the result of TPC in TA leaves in literature is 262.26 mg/g [49]. The total flavonoid content in TA leaves is 69.60 mgQE/100 g, while the result of total flavonoid content in TA leaves in literature is 76.15 mgQE/100 g [50]. Similarly, the inhibition of DPPH recorded is 39.01489%, while in literature, it was 45.28% [51]. The efficacy of nanoemulsion cream containing TA leaf extract was studied to heal acid-burn wounds induced with H_2_SO_4_ on the dorsum of the rabbits. A skin irritation test was conducted for any hypersensitivity reaction before applying nanoemulsion cream containing TA leaf extract on wounds. A chemical is deemed an irritant when it produces reversible skin lesions such as inflammation, which has healed partly or entirely until the observation period. It can cause irritated skin, ulcers, bleeding, and body scabs in rabbits. It was studied after applying 10 mL of cream on the rabbit’s left ear while the right was kept as a control. There was no sign of edema, and Escher or erythema were observed on the ear of the rabbit. This test showed the biocompatibility of nanoemulsion cream containing TA leaf extract with the skin, and it is non-immunogenic and safe.

The prepared nanoemulsion cream containing TA leaf extract was applied on the induced experimental animals compared with the commercial and untreated groups. Quench cream was used containing silver sulfadiazine as a commercial product. The size of the wounds was measured for 35 days. It was revealed that wounds treated with nanoemulsion cream containing TA leaf extract showed remarkable healing activity. During the second week of the trial, the wound treated with nanoemulsion cream containing TA leaf extract was reduced up to 60%. TA leaves contain various phytochemicals, which plays a major role as an antioxidant that prevents Oxidation, a chemical reaction that can lead to free radicals and chain reactions that can damage animals’ cells. After three weeks, that is 21 days, the acid-burn wound treated with nanoemulsion cream containing TA leaves showed complete healing, while the other acid-burn wounds treated with silver sulfadiazine showed complete healing after 28 days. Similarly, the wounds that were kept as control showed complete healing after 34 days. For histopathological studies, the skin tissues of the affected rabbits were taken at day 35. Then tissues were dipped in 10% formalin solution for 72 h and later were processed for histopathology study. The histological studies (epidermal thickness, collagen content and thickness of dermis) showed that the nanoemulsion containing TA accelerated the wound healing process better than the silver sulfadiazine. It was also revealed that nanoemulsion containing TA extract has significant cells attachment at the edge of the wound.

TA has been used for many testimonies since ancient times. Wound healing is one of the main indicators of the use of TA in many countries [21,22]. The results of this study give a free hand to the companies to start the formation of medicines in the form of ointments or cream. We hope a new burn ointment or cream can be used through herbal medicines with less unfavorable effects that will help in shortening the healing period thus it will decrease hypertrophic scar rate. Results showed that TA burn treatment is a cheap and useful herb. Significant pain relief was achieved within 24 h and overall healing period was achieved at twenty-one days as compared with the control and standard group. The nanoemulsion alone is difficult to apply on wound or skin due to low viscosity; therefore nanoemulsion was mixed with cream. The cream acts to carry or release the nanoemulsion. The cream base separately has no wound-healing effect or any activity. The mean size of the tualang honey-treated wound on day 21 was smaller in comparison to that of chitosan gel and hydrofibre silver treated groups. While nanoemulsion cream containing TA leaf extract resulted in a shorter period, 21.60 days, because the whole wound was treated, than the average healing time of the silver sulfadiazine group, 27.40 days, and positive control, 33.40 days. The average duration of healing was 18.16 and 32.68 days for the honey and SSD group, respectively, while the average healing duration in our trials for TA was 21.60 days and for silver sulfadiazine was 27.40 days. The mean size of the tualang honey-treated wounds was not statistically different than that of the chitosan gel or hydrofibre silver-treated wounds when the wounds were compared throughout the entire experiment (*p* > 0.05). However, comparing the mean wound size on day 21 alone revealed that the tualang honey-treated wounds were smaller in comparison with those of the chitosan gel and hydrofibre silver-treated groups [52,53]. 

## 5. Conclusions

Medicinal plants are an important natural resource and are thought to be potentially safe medicines. They have made significant contributions to reducing human misery throughout time. Herbal medications are used in rural primary health care systems, and in distant highland places, more than 70% of populations rely on folklore over the conventional medical system. TA is extensively utilized in traditional medicinal preparations due to its therapeutic activities. This is often planted in Pakistan and India’s plains since it is easy to grow from cuttings. The present study was proposed to investigate the therapeutic efficacy of nanoemulsion cream containing TA extract on acid-burn wounds. The plant leaves were collected and methanolic extract was prepared and incorporated into nanoemulsion. The nanoemulsions targeted drug delivery and acted as a vehicle for transdermal drug delivery, enhancing the drug’s wound-healing efficacy. The nanoemulsion prepared was characterized for zeta size, zeta potential, pH, viscosity, and stability. The nanoemulsion containing plant extract was converted into cream and evaluated for its efficacy against acid-burn wounds. Wound-healing experiment showed that nanoemulsion cream containing TA significantly decrease the healing duration compared with the standard medicine silver sulphadizine. From the above studies, it can be concluded that nanoemulsion cream containing TA extract has significant activity in promoting the healing of acid-burn wounds. Further studies should be conducted to substantiate our findings.

## Figures and Tables

**Table 1:** Material and their Quantity used in nanoemulsion.

S.No.	Material	Quantity
1	Water	10.50 gm.
2	Oil	3.50 gm.
3	TA leaf extract	3 gm.
4	Chitosan	200 mg

**Table 2:** Flavonoids and phenolic acids identified in T. aphylla by gradient HPLC.

Peak No	Compound	Reten. Time	Concentration (ppm)
1	Quercetin	2.907	17.11
4	Gallic acid	4.233	38.06
15	Caffeic acid	12.273	47.28
16	Vannilic acid	13.260	45.59
18	Benzoic acid	14.987	52.25
19	Syringic acid	16.307	15.96
20	p-Coumaric acid	17.907	32.38
22	m-Coumaric acid	19.800	13.76
27	Ferulic acid	25.260	64.18

**Table 3:** Identification of kaempferol in T. aphylla by isocratic HPLC.

Peak No	Compound	Reten. Time	Concentration (ppm)
3	Kaempferol	3.020	52.46

**Table 4:** Total Phenolic contents (TPC).

Sample	Sample Ab.	Blank Ab.			mgGAE/g ± SD
TA Leaf extract	1.201	0.098	1.103	220.6	220.6 ± 2.8

**Table 5:** Total Flavonoid Contents (TFC).

Sample	Absorbance		TFC in Sample (mgQE/100 g Extract)
TA Leaf extract	0.293	0.2645	69.60526 ± 1.5

**Table 6:** DPPH scavenging activity.

Sample	Blank	Sample			% Inhibition of DPPH
TA Leaf extract	1.746	1.414	0.332	0.190	39.01489

**Table 7:** Healing of wound at different time intervals.

	Control Group	Standard Group	Nanoemulsion Group
Day 0	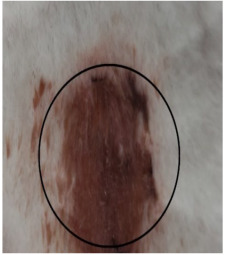	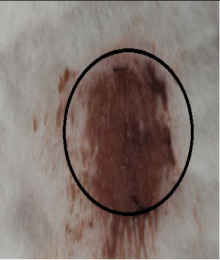	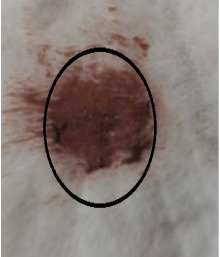
Day 7	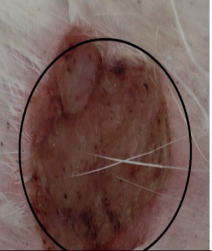	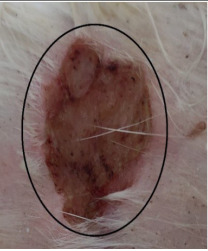	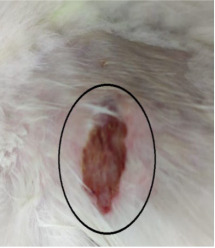
Day 14	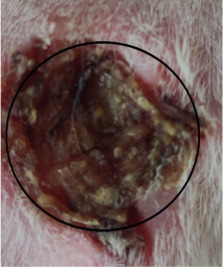	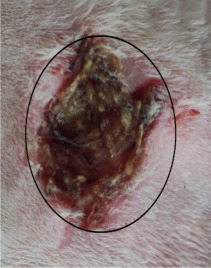	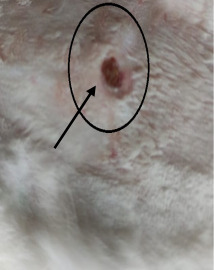
Day 21	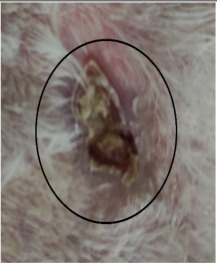	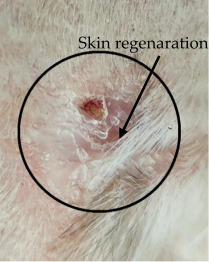	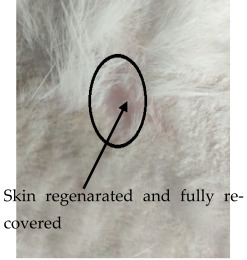

**Table 8:** Analysis of variance table for healing time (days).

Source	Degrees of Freedom	Sum of Squares	Mean Squares	F-Value	Prob.
Treatment	2	348.13	174.07	81.59 **	0.0000
Error	12	25.60	2.13		
Total	14	373.73			

** indicate (*p* < 0.01).

**Table 9:** Mean thickness of epidermis and dermis (μm), and collagen content percentage (%), * TA proved as significant (*p* < 0.05) statistically.

Groups	Mean Thickness (μm)		Collagen Content (%)
	Epidermis	Dermis	
TA	150.20 *	2185.8 *	93.85 *
Silver sulfadiazine	122.50	1802.3	85.72
Positive Control	15.17	1195.2	71.9

## Data Availability

Not applicable.
